# Nutritional Prehabilitation in Patients Undergoing Cystectomy: A Systematic Review

**DOI:** 10.3390/nu16111682

**Published:** 2024-05-29

**Authors:** Susy Dal Bello, Stefano Mancin, Sara Morales Palomares, Diego Lopane, Luca Di Gianfrancesco, Chiara Soligo, Tommaso Tarantino, Paolo Perdonò, Rodolfo Hurle, Bruno Bernardini, Federico Piccioni, Manuela Pastore, Alvarez Pellegrinelli, Angelo Porreca, Beatrice Mazzoleni

**Affiliations:** 1 Veneto Institute of Oncology IOV—IRCCS, 35128 Padua, Italy; susy.dalbello@iov.veneto.it (S.D.B.); chiara.soligo@iov.veneto.it (C.S.); tommaso.tarantino@iov.veneto.it (T.T.); paolo.perdono@iov.veneto.it (P.P.);; 2 IRCCS Humanitas Research Hospital, 20089 Rozzano, Italy; 3 Department of Pharmacy, Health and Nutritional Sciences (DFSSN), University of Calabria, 87036 Rende, Italy; 4 Santa Chiara Hospital, 38122 Trento, Italy; 5 Department of Biomedical Sciences, Humanitas University, 20072 Milan, Italy; beatrice.mazzoleni@hunimed.eu

**Keywords:** cystectomy, nutrition, prehabilitation, systematic review

## Abstract

Background/Aim: Nutrition is a key element of the prehabilitation process prior to surgery. The aim of this study was to identify the clinical pathways of nutritional prehabilitation before cystectomy. Methods: A systematic literature review was conducted in PubMed, the Cochrane Library, CINAHL, Scopus and the Web of Science databases. Quality and risk of bias assessment was conducted adhering to the JBI framework and evidence was evaluated according to the Oxford Centre for Evidence Based Medicine levels of evidence. Results: Out of 586 records identified, six studies were included. Among them, only two were randomized controlled trials. Immunonutrition has been shown to improve postoperative bowel function (3.12 vs. 3.74 days; RR 0.82; CI, 0.73–0.93; *p* = 0.0029) and decrease postoperative complications (−36.7%; *p* = 0.008) and readmission rates (−15.38%; *p* = 0.03). Furthermore, oral nutritional supplements combined with nutritional counseling demonstrated an accelerated recovery of bowel function (−1 day; *p* < 0.01), a reduction in the length of hospital stay (−1.75 days; *p* = 0.01), an improvement in handgrip strength (+6.8%, *p* < 0.001), an increase in bone mass (+0.3 kg, *p* = 0.04), and a better BMI value (+2.3%, *p* = 0.001). Conclusions: Nutritional prehabilitation demonstrates potential in enhancing postoperative outcomes following radical cystectomy. Oral supplements, immunonutrition, and counseling exhibit efficacy in improving postoperative results.

## 1. Introduction

Prehabilitation is an interdisciplinary approach involving the assessment of risk factors and implementing a tailored regimen of physical exercise, nutritional interventions, and holistic support to enhance a patient’s physiological and psychological reserves prior to surgery [1]. This proactive approach to health aims to enhance functional and psychological capabilities before surgery, thereby optimizing patient outcomes and overall well-being. In particular, prehabilitation interventions have garnered significant attention in the context of major surgical procedures [2]. Extensive research in this area has highlighted their efficacy in reducing overall and pulmonary morbidity, enhancing patients’ functional reserves, improving their quality of life, and positively impacting their psychological status [2,3]. By targeting physical and mental preparedness prior to surgery, prehabilitation interventions not only mitigate the risks associated with the procedure but also contribute to a smoother recovery process and better long-term prognosis, underscoring the importance of integrating prehabilitation strategies into routine clinical practice to maximize patient benefit and promote comprehensive perioperative care.

In the setting of patients undergoing cystectomy, prehabilitation is becoming an integral element of the clinical pathway [3]. Prehabilitation in oncology is defined as a process within the continuum of care that takes place between cancer diagnosis and the onset of acute treatment. This process includes nutritional, physical, and psychological assessments to establish a baseline functional level and pinpoint impairments. Targeted interventions are then employed to bolster the patient’s health, aiming to reduce the incidence and severity of present and future impairments [4]. This prepares the patient to better withstand the imminent physical challenges arising from surgical tissue trauma, reduced physical activity, and anxiety, which collectively contribute to a swift decline in functional capacity [5].

Recently, the importance of prehabilitation has gained increased recognition, especially for patients preparing for major bladder cancer surgery [6]. In response, scientific societies are incorporating prehabilitation into guidelines for radical cystectomies (RCs) and the preoperative period is considered an opportunity to proactively optimize bladder surgery [7]. Multimodal prehabilitation programs, which include physical exercises and nutritional supplements, have demonstrated themselves to be feasible and effective, leading to positive improvements in patients’ physical fitness and functional status. While rapid recovery pathways have become a standard in many surgical settings to enhance postoperative recovery, the nutritional status, a predictor of postoperative morbidity and mortality in oncology patients, is often under-considered in urology [8,9]. Understanding the profile of patients before major surgery could facilitate and refine the surgical outcomes. However, there is a gap in fully implementing this concept in urology. The impact of modifiable preoperative risk factors, such as nutritional and comorbidity status, in bladder cancer pathways remains incompletely explored. In patients with muscle-invasive bladder cancer (MIBC), neoadjuvant chemotherapy completes the treatment, with a further reduction in the patient’s reserve [10]. MBIC is a high-mortality disease, even when treated. RC is the frontline treatment for MBIC and for patients with non-muscle-invasive bladder cancer (NMBIC) carcinoma at high risk of disease progression. However, this major surgery implies a high risk of morbidity, mortality, and short- and long-term complications [11]. A comprehensive perioperative risk assessment before an RC should encompass objective measures of physiological age, physical function, nutrition, lean muscle mass, and frailty. Advocating for the use of standardized multidimensional tools for patients undergoing an RC can help identify and address modifiable risk factors through prehabilitation interventions [12].

Nutritional prehabilitation represents an important proactive approach focused on optimizing a patient’s nutritional status before surgery. This strategy is recommended because a good nutritional state can positively influence both resistance to infections and the post-surgery recovery by reducing the risk of complications and enhancing overall outcomes [13]. Given the positive results observed from nutritional prehabilitation in other cancer types [14], such as upper gastrointestinal tumors, there arises an urgent need to systematically assess its efficacy and implementation in the context of the cystectomy procedure.

### Systematic Review Objectives

The primary objective of this systematic review (SR) was to identify, through the existing literature, nutritional prehabilitation interventions aimed at preparing patients for an RC. Specifically, the SR aimed to determine: (1) The effectiveness of interventions in relation to nutritional status and anthropometric and muscle strength measurements; postoperative clinical outcomes, such as postoperative complications, infections, readmission rate; and inflammatory modulation. (2) The timing of the initiation and adherence of nutritional prehabilitation interventions. (3) The healthcare professionals involved in the nutritional prehabilitation process.

## 2. Materials and Methods

### 2.1. Review Methodology

This SR was conducted and reported according to the Preferred Reporting Items for Systematic Reviews and Meta-Analyses (PRISMA) Guidelines [15] and following the PRISMA checklist (Supplementary File S2).

### 2.2. Systematic Review Protocol Registration

The protocol of this SR was registered in the International Prospective Register of Systematic Reviews (PROSPERO) of the National Institute of Health Research available at https://www.crd.york.ac.uk/prospero/ (accessed on 19 January 2024) with the protocol registration number: CRD42024500386.

### 2.3. Formulation of the Research Question

The research question for this review was formulated using a PICO tool [16]. The PICO framework aids authors in formulating a research question for a review by focusing on four main components: the population or problem (P), the intervention of interest (I), the comparison (C), and the outcome (O). Tailoring this to our review aim, three primary aspects (PICOs) were integrated: P = Patients undergoing cystectomy; I = Nutritional prehabilitation interventions; C= standard of care or non-application of nutritional prehabilitation interventions; O = Efficacy of nutritional prehabilitation in patient candidates for cystectomy surgery, and its implications on post-operative outcomes; identification of healthcare professionals involved in the RC prehabilitation phase. 

### 2.4. Search Strategy

A comprehensive and systematic literature search was conducted between October and November 2023 to identify contemporary sources relevant to nutritional prehabilitation interventions in the context of cystectomy surgery. Scientific databases, including PubMed, the Cochrane Library, CINAHL, Scopus and the Web of Science, were meticulously scrutinized. To guarantee an exhaustive and holistic analysis, we further explored hospital-specific databases and other repositories of grey literature, which offer insights often not covered in conventional scientific publications. The search strategy encompassed terms such as “nutritional”, “prehabilitation”, and “cystectomy”, along with their relevant synonyms and related phrases. Boolean operators, namely AND and OR, were judiciously used to synthesize these terms, ensuring a broad yet focused search (Supplementary File S1). In the initial screening phase, two researchers (SDB and SM) independently assessed all titles and abstracts extracted from the electronic database searches. Utilizing EndNote 20 software (https://endnote.com/, accessed on 3 February 2024), duplicates and non-relevant records were systematically eliminated. In instances of discordance, a third researcher (DL) was consulted to facilitate consensus [17]. For those studies deemed potentially relevant in the initial screening, full-text articles were obtained. Each of these was subjected to a rigorous independent assessment by two researchers (SDB and SM) in alignment with predetermined eligibility criteria. In situations where the consensus was challenging, dialogues were initiated between the primary reviewers. If no agreement was reached, the adjudication of the third researcher (DL), who had not been previously involved with that specific paper, was sought to ensure an unbiased decision-making process.

### 2.5. Criteria and Process

Inclusion criteria comprised primary studies, written in English and published between 2010 and November 2023, focusing on nutritional prehabilitation interventions tailored for patients scheduled for cystectomy surgery. Conversely, the exclusion criteria were designed to systematically exclude book chapters, articles where the full-text versions were not accessible, editorials, and publications exhibiting low methodological quality. This rigorous selection process aims to uphold the scientific integrity and relevance of the sources incorporated into this SR.

### 2.6. Evaluation of Risk of Bias and Methodological Quality of Studies

The risk of bias and the methodological quality of the included articles were initially evaluated by two researchers (SDB and SM). Conflicts were resolved by the involvement of a third researcher (DL). To rigorously assess the methodological quality and relevance of the selected studies, we employed the Joanna Briggs Institute (JBI) Critical Appraisal Tools [18]. Recognized for their meticulousness in evaluating various research designs, these tools provided a structured framework to discern the reliability and applicability of each study. High-quality studies were identified based on a previous study [16], in which studies with a JBI score ≥ 70% were classified as having a high quality, those with a score between 69.9% and 50% as medium quality, and those with a score < 50% as low quality. 

### 2.7. Assessment of Evidence Certainty

The evidence certainty was evaluated using the framework established by the Oxford Centre for Evidence-Based Medicine (OCEBM) [19]. This framework categorizes research into five distinct levels of evidence based on study design and research quality. High-level studies, encompassing SRs of randomized controlled trials and superior individual trials, were conferred the first evidence tier. Conversely, research predominantly grounded in expert consensus or lacking empirical substantiation was categorized to the fifth level. Intermediate-level research, which includes but is not limited to less rigorous randomized controlled trials, cohort studies, and methodologies such as case series or case-control investigations, were allocated to the second, third, and fourth levels, respectively. Certain studies underwent a recalibration of their evidence level, either being elevated or diminished, influenced by parameters like methodological rigor, precision of findings, and the relevance of the results to the topic at hand.

### 2.8. Data Extraction 

Data from selected articles were extracted and reported in tables as follows: authors, year of publication, country, study design, populations, type of nutritional prehabilitation, and assessment of quality/bias. 

### 2.9. Synthesis Methods

The articles included in this SR were systematically categorized based on the treatments of the nutritional prehabilitation interventions, the optimal timing for their application, and the professionals involved. For each of these intervention classifications, the study methodologies and primary outcomes were articulated through a narrative synthesis. In this SR, despite recognizing the advantages of a meta-analysis, we concluded that a combined quantitative synthesis was impractical due to the diverse nature of the included studies, as explained in the Cochrane Handbook for Systematic Reviews of Interventions [20]. This diversity was marked by variations in intervention types and the methodologies for quantifying variable relationships, leading to a lack of consistency in both methodological and statistical aspects. As a result, we undertook a detailed narrative synthesis, adhering to the Synthesis Without Meta-analysis (SWiM) reporting guideline [21]. This technique was chosen for its effectiveness in transparently and robustly synthesizing varied quantitative data, in accordance with the PRISMA methodology [15]. To analyze the interactions between variables, the review utilized a “vote counting” method, as recommended by SWiM guidelines [21]. This method effectively addresses different measures of effects and was supported by the Cochrane Handbook for Systematic Reviews of Interventions [20]. We classified the results as “↑” indicating a positive effect (effective nutritional prehabilitation), “↓” for a negative effect (ineffective nutritional prehabilitation), and “=” for neutral effect (prehabilitation intervention with the same efficacy as standard treatment, if applicable). For studies presenting multiple effects data, each instance was evaluated individually. For a visual representation of effect directions and additional details of the studies, a Harvest plot was used [22], following the recommendations in the Cochrane Handbook [20]. The plot illustrates the source of information through references, uses bar heights to indicate sample sizes, shades bars to show the nature of the association (black for positive, gray for negative, and white for neutral effect), and labels to denote intervention types.

## 3. Results

### 3.1. Search Results

**Figure 1:**
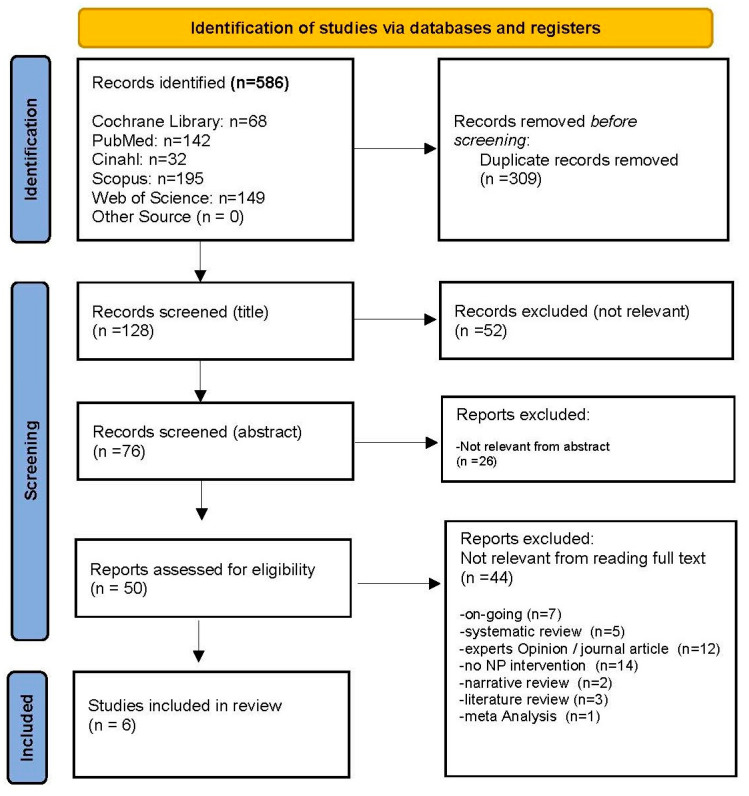
PRISMA Flow Diagram. NP = nutritional prehabilitation.

A total of 586 articles were identified through electronic database searches (Cochrane Library: 68, PubMed: 142, Cinahl: 32, Scopus: 195, and Web of Science: 149). After removing 309 duplicated articles and screening 128 titles, 76 articles were retained and evaluated for eligibility by reading the abstracts. Of these, 26 were judged not relevant, and 50 full texts were evaluated. Among these, 44 were further deleted as they did not meet our research criteria: they were not primary studies (*n* = 23), ongoing studies (*n* = 7), or did not report nutritional prehabilitation interventions (*n* = 14). Ultimately, the screening process included six studies in this SR (Figure 1). 

### 3.2. Characteristics of Studies, Population, and Interventions

This SR included six studies with a total sample of 619 patients (range 32–192). A significant portion of these studies, notably, were undertaken within American populations (567 patients). In terms of research design, there were two randomized controlled trials (RCTs) [23,24], one quasi-experimental study [25], one cohort study [26], one case-control study [27], and one cross-sectional study [28]. The prevalence of the studies included in the analysis exhibited commendable methodological quality and a minimal risk of bias, as evaluated through the application of the JBI critical appraisal tools, as detailed in Supplementary File S1. A comprehensive overview of the fundamental characteristics of the included studies is presented in Table 1. 

### 3.3. Immunonutrition 

**Figure 2:**
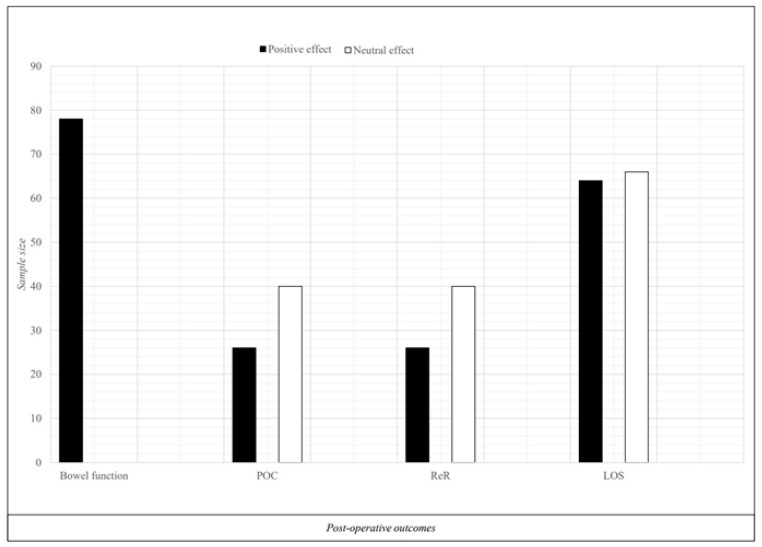
Effect of ONS and NC before radical cystectomy. Legend: POC = post-operative complication; ReR = readmission rate; LOS = length of stay; Aggregated data with statistical significance (*p* ≤ 0.05) are presented.

Studies that employed SIM interventions combined with preoperative immunonutrition yielded positive findings (Figure 2). Patel et al. [28] observed a significant improvement in bowel function one day post-surgery (3.12 vs. 3.74 days; relative risk 0.82; confidence interval, 0.73–0.93; *p* = 0.0029) compared to the control group, which followed only the standard enhanced recovery after surgery (ERAS) protocol without SIM administration. In another study [26], the use of SIM compared to the standard of care group, where SIM was not administered, resulted in a notable reduction in postoperative complications (POCs) (−36.7%; *p* = 0.008) and readmission rates (ReRs) (−15.38%; *p* = 0.03). Conversely, in a third study [23], SIM was compared with oral nutritional supplements (ONSs) (Kcal 360; protein 14 g). In both groups, administration for 5 days before the RC instructed patients to consume three cartons per day between meals. The results of this study demonstrated that in the SIM group, there was a 54.3% average increase in the balance between T helper lymphocytes of type 1 (Th1) and type 2 (Th2), while in the ONS group, the Th1–Th2 balance decreased by 4.8% (*p* < 0.027). Plasma interleukin-6 was 42.8% lower in the SIM group compared to the ONS group (*p* = 0.020). Additionally, plasma arginine levels remained stable in the SIM group, whereas the ONS group showed a 26.3% reduction (*p* = 0.0003). Lastly, in the study by Lyon et al. [24], SIM compared to ONS showed no significant differences between groups across the evaluated domains (*p* > 0.4). 

### 3.4. Oral Nutrition Supplements Combined with Nutritional Counseling 

**Figure 3:**
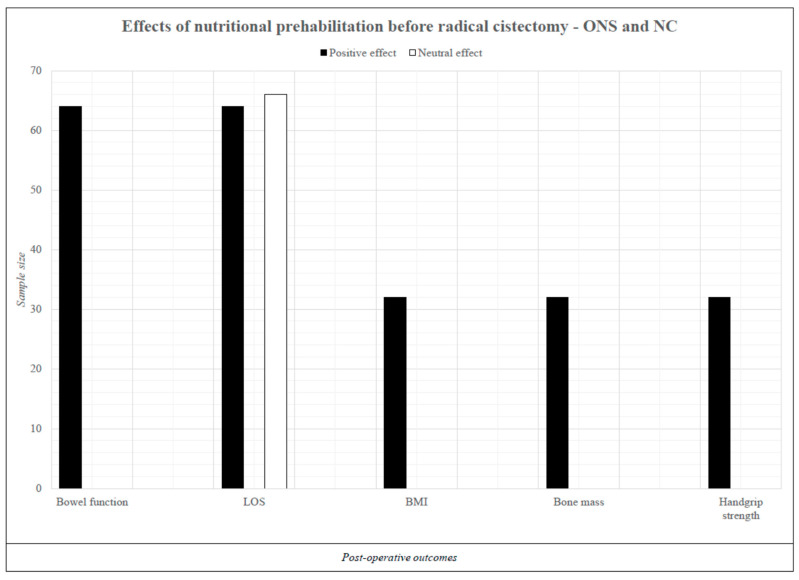
Effects of immunonutrition prior to radical cystectomy. Legend: ONS = oral nutritional supplement; NC = nutritional counselling; LOS = length of stay; BMI = body mass index. Aggregated data with statistical significance (*p* ≤ 0.05) are presented.

Two studies [25,27] employing ONSs in combination with nutritional counseling yielded positive outcomes (Figure 3). In the study by Aldhaam et al. [27], an ERAS prehabilitation pathway involving nutritional counseling 4 weeks before the RC coupled with ONS administration 4 days before the RC (specific portion/day not specified), compared to the control group (CG) receiving only nutritional counseling, demonstrated accelerated recovery of the bowel function (1 day faster, *p* < 0.01) and a reduction in length of hospital stay by 1.75 days (*p* = 0.01). In a second study [25], involving a sample of 32 patients undergoing an RC, nutritional counseling combined with ONS administration (300 kcal, 12.5 g of protein, or in diabetic patients, 330 kcal, 16.5 g of protein) in two portions/day for 2 weeks before the RC, resulted in improvements in handgrip strength (+6.8%, *p* < 0.001), bone mass (+0.3 kg, *p* = 0.04), and better BMI value (+2.3%, *p* = 0.001). 

### 3.5. Protocols and Timing of Nutritional Prehabilitation Interventions

#### 3.5.1. Immunonutrition

The timing of and adherence to nutritional prehabilitation interventions play a crucial role in optimizing patient outcomes before an RC. Patel et al. [28] elaborated a SIM protocol consisting of ω-3, arginine, and nucleotides, providing 200 kcal and 18 gr. of protein per portion. This intervention was administered three times daily for 5 days preceding the RC. A further study [23,26] adopted a similar approach, employing a standard immunonutrition supplement containing ω-3, arginine, and nucleotides, providing 341 kcal and 18 gr. of protein per portion three times daily for 7 days preceding the RC. Lastly, Lyon et al. [24] also utilized the same immunonutrition four times daily for 5 days before the RC. These interventions underscore the importance of precise timing and the patient’s compliance with SIM protocols in the preoperative period, aiming to enhance patient nutritional status and optimize surgical outcomes in individuals undergoing an RC [23,24,26,28]. Furthermore, two studies extended the nutritional intervention into the postoperative phase. Specifically, the study by Hamilton-Reeves et al. [23] continued the administration of immunonutrition at the same dosage as the preoperative phase for an additional 5 days, while in the study by Cozzi et al. [26], the group receiving immunonutrition continued postoperative administration at a reduced dosage for 7 days (twice daily).

Adherence to these protocols varied between studies, with Patel et al. [28] reporting a 100% adherence rate, Cozzi et al. [26] and Hamilton-Reeves et al. [23] not providing adherence data, and Lyon et al. [24] reporting an 83% adherence rate.

#### 3.5.2. Oral Nutrition Supplements Combined with Nutritional Counseling

ONS interventions combined with nutritional counseling have shown positive results in prehabilitation. Aldhaamet al. [27] employed ONSs in addition to nutritional counseling, incorporating unspecified nutritional supplements and education sessions to assess nutritional status and needs 5 days before the RC. Similarly, Jensen et al. [25] utilized ONSs (standard 300 kcal, 12.5 g of protein, or in diabetic patients 330 Kcal, 16.5 g of protein) along with nutritional counseling, administering these twice daily for 2 weeks before the RC, accompanied by nutritional education and assessment. These interventions, coupled with personalized nutritional counseling, aim to address individual patient needs and optimize nutritional status before the RC, potentially improving surgical outcomes and patient recovery. Analyzing the study by Aldhaam et al. [27], the nutritional intervention was extended not only during the preoperative phase but also encompassed the postoperative phase through the administration of the same nutritional formulation provided in the preoperative phase, specifically upon resumption of the oral fluid intake. In addition to oral supplementation, the nutritional protocol included the administration of a 1000 mL saline solution on postoperative days 2, 5, and 7.

Adherence to ONS data was not reported by Aldhaam et al. [27], while Jensen et al. [25] reported a commendable adherence rate of 81.2%, highlighting the importance of patient compliance in achieving the desired outcomes of the ONSs combined with nutritional counseling.

### 3.6. Healthcare Professionals Involved

Aldhaam et al. [27] highlighted the role of a nutritionist in their study, emphasizing the importance of specialized nutritional counselling in the prehabilitation process. In a second study [25], the involvement of a multidisciplinary team comprising one surgeon and two clinical nurse specialists (CNSs) was reported, indicating a collaborative approach to patient care. However, four studies [23,24,26,28] did not specify the healthcare professionals directly involved in the interventions or the prehabilitation process. A summary of the nutritional prehabilitation interventions identified is shown in Table 2.

### 3.7. Summary of the Evidence

This SR, encompassing six studies, [23,24,25,26,27,28] examines the impact of nutritional prehabilitation interventions on outcomes following radical cystectomy, revealing diverse effects on postoperative parameters. Among the interventions, SIM emerges as a significant strategy for enhancing postoperative bowel function. Patel et al. [28] demonstrated notable improvements in bowel function 1 day post-surgery among participants receiving SIM compared to the ONSs. Additionally, Cozzi et al. [26] reported decreased postoperative complications and respiratory complications with perioperative immunonutrition, indicating its potential in reducing surgical morbidity. In support, studies employing the ONSs combined with nutritional counseling highlighted the benefits of a multimodal approach. In one study, [27] accelerated bowel function recovery and reduced length of hospital stay were observed in patients receiving nutritional counseling plus ONSs. Similarly, a second study [25] documented improvements in physical parameters, such as handgrip strength, bone mass, and BMI reduction with the combined intervention. Collectively, all studies [23,24,25,26,27,28] included support the importance of tailored nutritional interventions in preoperative care, aiming to enhance patient outcomes and improve surgical recovery following an RC.

## 4. Discussion

Nutritional prehabilitation interventions are increasingly recognized as integral components in optimizing outcomes for patients undergoing an RC, a complex surgical procedure for bladder cancer, associated with significant postoperative morbidity and mortality. Given the challenges posed by an RC, preoperative optimization becomes crucial for enhancing patient recovery and reducing surgical complications. In response to this recognition, numerous studies have delved into the efficacy of various nutritional interventions, encompassing SIM protocols and ONSs combined with nutritional counseling [23,24,25,26,27,28]. The multifaceted nature of RC surgery necessitates a tailored approach to preoperative optimization, addressing not only nutritional deficiencies but also the broader physiological and metabolic challenges posed by the procedure [29,30]. This systematic review highlights the diverse strategies employed across studies, reflecting the evolving landscape of prehabilitation interventions in the context of an RC. These interventions span a spectrum of modalities, ranging from oral supplements to immunonutrition and personalized counseling, underscoring the nuanced and individualized nature of preoperative care in this patient population [20,21,22,23,24,25]. Nutrition assumes a critical role before surgery, especially among patients who may have undergone neoadjuvant chemotherapy treatments. Such individuals often face nutritional challenges like appetite loss, nausea, vomiting, and alterations in taste perception [31,32], underscoring the importance of addressing nutritional needs as part of a comprehensive and targeted preoperative management. As elucidated by two studies, [33,34] oral supplements and immunonutrition emerge as promising treatments for mitigating postoperative complications and expediting recovery in RC patients.

We must also consider the role of artificial nutrition in nutritional prehabilitation interventions, as it can significantly improve the nutritional status and functional capacity of patients undergoing cystectomy procedures. This is particularly important in cases where patients have difficulty ingesting nutrients orally, such as due to dysphagia, gastrointestinal obstructions, or other disorders limiting absorption. In such circumstances, in addition to enteral nutrition infused through a nasogastric tube or nutritional stoma, the role of parenteral nutrition must be considered. Parenteral nutrition can provide comprehensive nutritional support directly through the circulatory system, bypassing the gastrointestinal tract, and may be vital for maintaining a nutritional balance in patients who cannot consume food orally, or who have limited intestinal absorption capacity or complications that preclude feeding through the digestive tract. It is also worth noting that to date, there are no studies that have applied such interventions in nutritional prehabilitation before cystectomy procedures. However, evidence is available regarding nutritional prehabilitation interventions using artificial nutrition prior to major surgical procedures [35].

Furthermore, prehabilitation’s efficacy extends beyond bladder cancer, with studies of colorectal, head and neck, lung cancer and in major surgery [2] reporting reductions in length of the hospital stay and decreased postoperative complication rates [3,36,37,38]. Findings from the colorectal cancer study and the major surgery [2,36] highlight the multifaceted benefits of prehabilitation, encompassing enhanced functional capacity, reduced postoperative complications, shortened length of hospital stay, and improved quality of life. Drawing parallels with insights from the systematic review on head and neck cancer, which underscores the importance of combining nutritional and physical prehabilitation interventions, there is a promising convergence toward elevating patient management outcomes in the context of an RC. However, while the results of this review support the significant benefits of prehabilitation in terms of postoperative complications, infections, and readmission rate, determining the precise timing of administration, dosage, composition, and adherence of nutritional supplements remains challenging. Therefore, it is crucial to delineate how nutritional prehabilitation pathways often exhibit heterogeneity; in particular, considering variations in dosages, timing of the initiation, and notably, the extension of immunonutrition [23,26] or ONS [27] interventions into the postoperative phase in some studies [23,26,27], with variable durations. Moreover, one must consider how the efficacy of this prolonged nutritional intervention approach remains uncertain, primarily due to limited or heterogeneous sample sizes and diverse outcomes analyzed in the included primary studies. This underscores the importance of initiating studies, such as RCTs, that include homogeneous samples with larger sample sizes and well-defined pathways, as emphasized by additional previously published studies [39,40,41].

Furthermore, this systematic review underscores the importance of adopting a comprehensive and integrated multidisciplinary approach to nutritional prehabilitation, in line with broader initiatives aimed at improving patient outcomes and quality of life throughout the entire oncological care continuum. Although the number of studies specifically examining the professionals involved in the care process is limited, the studies included in this review [25,27] emphasize its multidisciplinary nature. Physicians, surgeons, nutritionists, and other specialized professionals, such as clinical nurse specialists (CNSs) and speech therapists, are essential in nutritional prehabilitation programs. Surgeons provide essential medical expertise and oversee the surgical aspect of care, ensuring optimal surgical outcomes, supported by dieticians and nutritionists who provide crucial dietary guidance and offer personalized nutritional support tailored to the specific needs of each patient [42,43]. CNSs play a fundamental role by providing comprehensive patient education, coordinating nursing care between various healthcare providers, and addressing psychosocial factors that may impact nutritional well-being [44], also involving professionals such as psychologists. This collaborative effort ensures that patients receive holistic and integrated care, addressing not only their surgical needs but also their nutritional and psychosocial well-being, thereby optimizing their overall health outcomes and surgical success.

### Limitations

However, it is important to recognize some limitations of the review. The presence of a limited number of studies and relatively small sample sizes in some of the included studies raises concerns regarding the generalizability and potential bias of their results. The variability in clinical–nutritional pathways and protocols across studies introduces heterogeneity into the analysis, making it difficult to draw definitive conclusions on the most effective approach to prehabilitation. Additionally, variations in the study design and methodology among the included studies may impact the overall quality of evidence.

## 5. Conclusions

Current evidence suggests a promising role for nutritional prehabilitation interventions in optimizing postoperative outcomes for patients undergoing radical cystectomy. The use of oral nutritional supplements, immunonutrition, and nutritional counseling has shown some efficacy in improving postoperative outcomes, modulating the inflammatory response and enhancing nutritional status. Additionally, nutritional interventions should be personalized. Identifying risk factors associated with poor nutrition through a comprehensive patient assessment could further individualize interventions. However, further research is needed to refine and optimize these interventions. Future studies should address the intrinsic limitations identified in the existing literature, including larger sample sizes, to overcome the heterogeneity of interventions identified in this review.

## Figures and Tables

**Table 1:** Characteristics of the included studies.

Author	Year	Country	Design	Populations (*n*)	NP Intervention	Quality/Bias
Patel et al. [28]	2022	USA	Cross-sectional study	(*n* = 170) IG = 78 CG = 92	SIM-carbohydrates loading (IG) ONS (CG)	+++/High
Cozzi et al. [26]	2021	Italy	Cohort study	(*n* = 52) IG = 26 CG = 26	SIM (IG) SOC (CG)	+++/High
Aldhaam et al. [27]	2020	USA	Case control study	(*n* = 192) IG = 64 CG = 128	Nutritional Counseling-ONS (IG) Nutritional counseling (CG)	+++/High
Jensen et al. [25]	2019	USA	Quasi-experimental study	(*n* = 32)	Nutritional Counseling-ONS (IG)	++/Medium
Hamilton-Reeves et al. [23]	2018	USA	RCT	(*n* = 29) IG = 14 CG = 15	SIM (IG) ONS (CG)	+++/High
Lyon et al. [24]	2017	USA	RCT	(*n* = 144) IG = 40 CG = 104	SIM (IG) SOC (CG)	++/Medium

Legend: NP = nutritional prehabilitation; SIM = specialized immunonutrition; ONS = oral nutritional supplements; IG = intervention group; CG = control group; SOC = standard of care; RCT = randomized controlled trial. Quality/Bias according to JBI Critical Appraisal Tools and Mancin et al. [16]: High = +++; Medium = ++.

**Table 2:** Summary of Nutritional Prehabilitation Interventions.

Author	NP (IG) Interventions	NP (IG) Characteristics	NP (IG) Timing	Healthcare Professional	Outcomes	Results	(%) NP Adherence	OCEBM Evidence Level
Patel et al. [28]	SIM and carbohydrates loading (IG)	- ω-3, arginine, and nucleotides; calories 200 kcal, protein 18 g	3 portions/day for 5 days before RC	NA	POC rates	↑ Bowel function: (−1 day = 0.001)	100%	3
						= POC (−7.8% *p* = 0.36)		
						↑ IC (−12.9% *p* = 0.53)		
					LOS	↑ LOS (6 vs. 5 days *p* = 0.12)		
	ONS (CG)	- maltodextrin; calories 200 kcal, protein 0 g	The night before RC and 2 h prior to RC.					
					ReR	↑ ReR (−6.9% *p* = 0.34)		
Cozzi et al. [26]	SIM (IG)	- ω-3, arginine, and nucleotides; calories 341 kcal, protein 18 g.	3 portions/day for 7 days before RC	NA	POC rates	↑ POC (−36.7% *p* = 0.008)	NA	3
					ReR	↑ ReR (−15.38% *p* = 0.03)		
	SOC (CG)					= LOS Median LOS: 10 days (IG = IQR: 9–16; CG = IQR: 9–13; *p* = 0.39).		
					LOS			
Aldhaam et al. [27]	Nutritional Counseling and ONS (IG)	- Nutritional education, assessment of nutritional status/needs	4 weeks before RC	Nutritionist	POC rates	↑ Bowel function: 1 day faster, *p* < 0.01	NA	3
	Nutritional counseling (CG)	- Nutritional supplements (not specified)	5 days before RC (portion/day not specified)		LOS	↑ LOS (−1.75 days, *p* = 0.01)		
Jensen et al. [25]	Nutritional Counseling and ONS (IG)	- Nutritional education, assessment of nutritional status/needs	2 portions/day for 2 weeks before RC	Surgeon CNS	Baseline functional	↑ Handgrip strength (+6.8% *p* < 0.001)	81.2%	2
						↑ Bone mass (+0.3 kg *p* = 0.04)		
		- ONS 300 kcal, protein 12.5 g/diabetic patients 330 kcal, protein 16.5 g.			Nutritional status			
						↑ BMI reduction (CG: −2.3% *p* = 0.001)		
Hamilton-Reeves et al. [23]	SIM (IG)	- ω-3, arginine, and nucleotides; calories 341 kcal, protein 18 g.	3 portions/day for 5 days before RC	NA	Inflammatory modulation	↑ Th1-Th2 balance (SIM = +54.3%; ONS = −4.8%)	NA	2
						↑ Plasma interleukin-6 (lower in the SIM group *p* = 0.022)		
	ONS (CG)	ONS 360 kcal; protein 14 g.						
						= Plasma arginine (*p* = 0.002)		
Lyon et al. [24]	SIM (IG)	- ω-3, arginine, and nucleotides; calories 341 kcal, protein 18 g.	SIM 4 portions, 5 days before RC	NA	POC rates	= no difference between IG vs. CG in any domains evaluated (*p* > 0.4)	83%	1
					LOS			
	SOC (CG)				ReR			

Legend: NP = nutritional prehabilitation; RC = radical cystectomy; NA = not applicable; CNS = clinical nurse specialist; POC = post-operative complication; IC = infections complication; ReR = readmission rate; SIM = specialized immunonutrition; ONS = oral nutritional supplements; IG = intervention group; CG = control group; ↑: IG positive outcome; =: IG neutral outcome; SOC = standard of care; LOS = length of stay; BMI = body mass index. Th1 = Helper T cell 1; Th2 = Helper T cell 2; IQR = interquartile range; Evidence certainty according to Oxford Centre for Evidence-Based Medicine (OCEBM).

## Data Availability

All available data are provided as Supplementary Materials.

## Supplementary Materials

The following supporting information can be downloaded at: https://www.mdpi.com/article/10.3390/nu16111682/s1, Supplementary File S1: search strategy. Supplementary File S2: PRISMA 2020 Checklist.
