# Supplementary material for: Nutritional Prehabilitation in Patients Undergoing Cystectomy: A Systematic Review
Source: Nutrients. 2024 May 29;16(11):1682. doi: 10.3390/nu16111682 (PMC11174884; doi:10.3390/nu16111682)
Supplement: Supplementary file 1 [file nutrients-16-01682-s001.zip › Supplementary File S1.pdf]

Supplementary File S1

Search Strategy

ID	Cochrane Library	Results
#1	Cystectomies OR Cystectomy OR “bladder cancer*” OR “bladder neoplasm*” OR “bladder Tumor*”	
#2	"Nutrition Assessment*" OR "Nutritional Assessment*" OR "Nutrition" OR "Nutritional"	
#3	Prehabilitation OR Prehabilitations OR Pre-habilitation OR Pre-habilitations OR Preoperative OR "Pre-operative" OR "Pre operative" OR Conditioning OR Exercise*	
**#4**	**#1 AND #2 AND #3**	**68**
	**Pubmed**	**Results**
#1	Cystectomies OR Cystectomy OR “bladder cancer*” OR “bladder neoplasm*” OR “bladder Tumor*”	
#2	"Nutrition Assessment*" OR "Nutritional Assessment*" OR "Nutrition" OR "Nutritional"	
#3	Prehabilitation OR Prehabilitations OR Pre-habilitation OR Pre-habilitations OR Preoperative OR "Pre-operative" OR "Pre operative" OR Conditioning OR Exercise*	
**#4**	**#1 AND #2 AND #3**	**142**
**ID**	**Cumulative Index Of Nursing And Allied Health Literature (CINAHL)**	**Results**
#1	Cystectomies OR Cystectomy OR “bladder cancer*” OR “bladder neoplasm*” OR “bladder Tumor*”	
#2	"Nutrition Assessment*" OR "Nutritional Assessment*" OR "Nutrition" OR "Nutritional"	
#3	Prehabilitation OR Prehabilitations OR Pre-habilitation OR Pre-habilitations OR Preoperative OR "Pre-operative" OR "Pre operative" OR Conditioning OR Exercise*	
**#4**	**#1 AND #2 AND #3**	**32**
**ID**	**SCOPUS**	
#1	Cystectomies OR Cystectomy OR “bladder cancer*” OR “bladder neoplasm*” OR “bladder Tumor*”	
#2	"Nutrition Assessment*" OR "Nutritional Assessment*" OR "Nutrition" OR "Nutritional"	
#3	Prehabilitation OR Prehabilitations OR Pre-habilitation OR Pre-habilitations OR Preoperative OR "Pre-operative" OR "Pre operative" OR Conditioning OR Exercise*	

**#4**	**#1 AND #2 AND #3**	**195**
**ID**	**Web of Science**	
#1	Cystectomies OR Cystectomy OR “bladder cancer*” OR “bladder neoplasm*” OR “bladder Tumor*”	
#2	"Nutrition Assessment*" OR "Nutritional Assessment*" OR "Nutrition" OR "Nutritional"	
#3	Prehabilitation OR Prehabilitations OR Pre-habilitation OR Pre-habilitations OR Preoperative OR "Pre-operative" OR "Pre operative" OR Conditioning OR Exercise*	
**#4**	**#1 AND #2 AND #3**	**149**
	**TOTAL ARTICLES FOUND**	**586**

**INCLUSION / EXCLUSION CRITERIA**		
	**DUPLICATES**	**309**
	**TOTAL (DUPLICATES EXCLUDED)**	**277**
	**AFTER TITLE ABSTRACT READ**	**128**
	**AFTER FULL TEXTS READ**	**50**
	**RECORDS INCLUDED:**	**6**
	**RECORDS EXCLUDED:**	**44**

	**RECORDS INCLUDED:**	
	n. 3-7-14-20-33-42	
	**RECORDS EXCLUDED:**	**REASON:**
7	n.1- 36-37-38-39-40-41	on-going
5	n. 2 -8-16-22-44	systematic review
12	n.4-5-6-10-17-24-25-26-27-31-32-46	Experts Opinion / journal article
14	n. 9-11-12-13-15-19-21-23-28-29 -30 -34-45-50	no NP intervention
2	n. 18-47	narrative review
3	n. 35-48-49	literature review

1	n. 43	Meta Analysis

REFERENCES INCLUDED / EXCLUDED:

1. Akdemir, E., Sweegers, M. G., Vrieling, A., Rundqvist, H., Meijer, R. P., Leliveld-Kors, A. M., Van Der Heijden, A. G., Rutten, V. C., Koldewijn, E. L., Bos, S. D., & et al. (2023). EffectiveNess of a multimodal preHAbilitation program in patieNts with bladder canCEr undergoing radical cystectomy: protocol of the ENHANCE multicentre randomised controlled trial [Journal article]. BMJ Open, 13(3). https://doi.org/10.1136/bmjopen-2022-071304

2. Alam, S. M., Michel, C., Robertson, H., Camargo, J. T., Linares, B., Holzbeierlein, J., & Hamilton-Reeves, J. M. (2021). Optimizing Nutritional Status in Patients Undergoing Radical Cystectomy: A Systematic Scoping Review. Bladder Cancer, 7(4), 449-461. https://doi.org/10.3233/blc-200428

3. Aldhaam, N. A., Elsayed, A. S., Hussein, A. A., Siam, A., Osei, J., Jing, Z., Babar, T., Graton, M., Kurtz, T., Johnson, T., & et al. (2021). Impact of Perioperative Multidisciplinary Rehabilitation Pathway on Early Outcomes after Robot-assisted Radical Cystectomy: a Matched Analysis [Journal article]. Urology, 147, 155-161. https://doi.org/10.1016/j.urology.2020.05.113

4. Aning, J., Neal, D., Driver, A., & McGrath, J. (2010). Enhanced recovery: From principles to practice in urology [Note]. BJU International, 105(9), 1199-1201. https://doi.org/10.1111/j.1464-410X.2010.09249.x

5. Campodonico, F., & Parodi, D. (2013). Re: Lambert et al.: Using preoperative albumin levels as a surrogate marker for outcomes after radical cystectomy for bladder cancer (Urology 2013;81:587-592) [Letter]. Urology, 81(6), 1382. https://doi.org/10.1016/j.urology.2013.02.007

6. Claps, F., Pavan, N., Maresma, M. C. M., Mazzon, G., del Grappa, B., Ramirez-Backhaus, M., Boltri, M., Soria, F., Massanova, M., D'Andrea, D., Traunero, F., Marra, G., Liguori, G., Gontero, P., Shariat, S. F., Briones, J. R., Celia, A., & Trombetta, C. (2020). IMPACT OF PREOPERATIVE CONTROLLING NUTRITIONAL STATUS (CONUT) SCORE ON PERIOPERATIVE MORBIDITY AND SURVIVAL OUTCOMES IN PATIENTS WITH BLADDER CANCER TREATED WITH RADICAL CYSTECTOMY: A MULTICENTER ANALYSIS. Journal of urology, 203, E1278-E1278. <Go to ISI>://WOS:000527010301174

7. Cozzi, G., Musi, G., Milani, M., Jemos, C., Gandini, S., Mazzoleni, L., Salé, E. O., & de Cobelli, O. (2021). Impact of perioperative immunonutrition on complications in patients undergoing radical cystectomy. In Enhanced Recovery After Surgery: Perspectives, Protocols and Efficacy (pp. 65-83). https://www.scopus.com/inward/record.uri?eid=2-s2.0-85109466350&partnerID=40&md5=e62f53e71fed473fe6758f73cac87482

8. Cui, H. W., Turney, B. W., & Griffiths, J. (2017). The Preoperative Assessment and Optimization of Patients Undergoing Major Urological Surgery [Review]. Current Urology Reports, 18(7), Article 54. https://doi.org/10.1007/s11934-017-0701-z

9. Djaladat, H., Bruins, H. M., Miranda, G., Cai, J., Skinner, E. C., & Daneshmand, S. (2014). The association of preoperative serum albumin level and American Society of Anesthesiologists (ASA) score on early complications and survival of patients undergoing radical cystectomy for urothelial bladder cancer [Article]. BJU International, 113(6), 887-893. https://doi.org/10.1111/bju.12240

10. Durai, P., Lin, H. Z. J., Bhaumik, J., & Tong, P. S. Y. (2022). Prehabilitation for Surgery in Urology, Urogynaecology, and Gynaecological Oncology. In Prehabilitation for Cancer Surgery (pp. 295-310). https://doi.org/10.1007/978-981-16-6494-6_14

11. Garg, T., Chen, L. Y., Kim, P. H., Zhao, P. T., Herr, H. W., & Donat, S. M. (2014). Preoperative serum albumin is associated with mortality and complications after radical cystectomy. BJU International, 113(6), 918-923. https://doi.org/10.1111/bju.12405

12. Giese, M., Butea-Bocu, M., Huber, J., & Groeben, C. (2023). Prähabilitation bei radikaler Zystektomie. Die Urologie, 62(10), 1034-1040. https://doi.org/10.1007/s00120-023-02172-8

13. Gregg, J., Salem, S., Chang, S., Clark, P., Cookson, M., Davis, R., Stimson, C. J., Aghazadeh, M., Smith, J., & Barocas, D. (2010). EFFECTS OF PRE-OPERATIVE NUTRITIONAL DEFICIENCY ON 90-DAY MORTALITY AND OVERALL SURVIVAL IN PATIENTS UNDERGOING RADICAL CYSTECTOMY FOR BLADDER CANCER. Journal of urology, 183(4), E704-E705. https://doi.org/10.1016/j.juro.2010.02.1754

14. Hamilton-Reeves, J. M., Stanley, A., Bechtel, M. D., Yankee, T. M., Chalise, P., Hand, L. K., Lee, E. K., Smelser, W., Mirza, M., Wyre, H., & et al. (2018). Perioperative Immunonutrition Modulates Inflammatory Response after Radical Cystectomy: results of a Pilot Randomized Controlled Clinical Trial [Journal article]. Journal of urology, 200(2), 292-301. https://doi.org/10.1016/j.juro.2018.03.001

15. Hendri, A. Z., Khalilullah, S. A., & Aditya, G. A. (2021). A preliminary outcome of modified enhanced recovery protocol versus standard of care in radical cystectomy: an Indonesian experience [Article]. African Journal of Urology, 27(1), Article 111. https://doi.org/10.1186/s12301-021-00213-2

16. Hijazi, Y., Gondal, U., & Aziz, O. (2017). A systematic review of prehabilitation programs in abdominal cancer surgery [Review]. International Journal of Surgery, 39, 156-162. https://doi.org/10.1016/j.ijsu.2017.01.111

17. Holzbeierlein, J. M. (2022). Perioperative optimization of the genitourinary oncology patient [Review]. Urologic Oncology: Seminars and Original Investigations, 40(11), 487-488. https://doi.org/10.1016/j.urolonc.2021.04.019

18. Hupe, M. C., Kramer, M. W., & Merseburger, A. S. (2015). Preoperative and Modifiable Factors to Lower Postoperative Complications After Radical Cystectomy [Review]. Current Urology Reports, 16(4). https://doi.org/10.1007/s11934-015-0493-y

19. Jensen, B., Laustsen, S., Jensen, J., Borre, M., Petersen, A., Jensen, B. T., Jensen, J. B., & Petersen, A. K. (2016). Exercise-based pre-habilitation is feasible and effective in radical cystectomy pathways-secondary results from a randomized controlled trial. Supportive care in cancer, 24(8), 3325-3331. https://doi.org/10.1007/s00520-016-3140-3

20. Jensen, B. T., Jensen, J. B., Love-Retinger, N., Bowker, M., Retinger, C., & Dalbagni, G. (2019). Implementing a Multimodal Prehabilitation Program to Radical Cystectomy in a Comprehensive Cancer Center: A Pilot Study to Assess Feasibility and Outcomes. Urol Nurs, 39(6), 303-313. https://doi.org/10.7257/1053-816x.2019.39.6.303

21. Jensen, B. T., Jensen, K. E.-M., Lash, T. L., Laustsen, S., Petersen, A. K., & Borre, M. (2012). 28 The Impact of Pre-operative Nutritional Risk on Survival Following Radical Cystectomy. European Journal of Oncology Nursing, 16, S11-S11. https://doi.org/10.1016/S1462-3889(12)70043-1

22. Jensen, B. T., Lauridsen, S. V., & Jensen, J. B. (2020). Optimal delivery of follow-up care after radical cystectomy for bladder cancer [Review]. Research and Reports in Urology, 12, 471-486. https://doi.org/10.2147/RRU.S270240

23. Jensen, B. T., Laustsen, S., Jensen, J., Petersen, A. K., & Borre, M. (2016). Exercise-based prehabilitation is feasible and effective in radical cystectomy pathways-secondary results from a randomized controlled trial [Journal article; Conference proceeding]. European urology, supplements, 15(3), e332. https://www.cochranelibrary.com/central/doi/10.1002/central/CN-01142799/full

24. Karl, A., Buchner, A., Becker, A., Grimm, T., & Stief, C. G. (2013). Results of a prospective study on a new fast track regimen for patients undergoing radical cystectomy for bladder cancer [Journal article; Conference proceeding]. European urology, supplements, 12(1), e923. https://www.cochranelibrary.com/central/doi/10.1002/central/CN-01057553/full

25. Kassouf, W., Minnella, E., Awasthi, R., Ferreira, V., Aprikian, A., Tanguay, S., & Carli, F. (2018). Prehabilitation for patients undergoing cystectomy: preliminary analysis of a single-center, randomized controlled trial [Journal article; Conference proceeding]. Journal of urology, 199(4), e622. https://www.cochranelibrary.com/central/doi/10.1002/central/CN-01568992/full

26. Khan, A., Platek, M., Rehman, S., Din, R., Shi, Y., Azzouni, F., Wilding, G., & Guru, K. (2013). PROSPECTIVE EVALUATION OF PREOPERATIVE NUTRITIONAL STATUS IN PATIENTS UNDERGOING ROBOT-ASSISTED RADICAL CYSTECTOMY. Journal of urology, 189(4), E669-E669. https://doi.org/10.1016/j.juro.2013.02.3177

27. Kukreja, J. B., Seif, M., Lozano, M., Wang, X., Hwang, H., Kamat, A., Dinney, C., & Navai, N. (2019). Preoperative immunonutrition in patients treated with radical cystecotmy, results from a phase 1, "oral immunonutrition to enhance recovery after surgery" feasibility trial [Journal article; Conference proceeding]. Journal of urology, 201(4), e537. https://doi.org/10.1097/01.JU.0000556085.15762.2a

28. Lee, G., Patel, H. V., Srivastava, A., & Ghodoussipour, S. (2022). Updates on enhanced recovery after surgery for radical cystectomy [Review]. Therapeutic Advances in Urology, 14. https://doi.org/10.1177/17562872221109022

29. Li, S., Zhang, D., Zeng, S., Wu, T. J., Wang, Y. C., Zhang, H., Wang, B., & Hu, X. P. (2021). Prognostic Value of Preoperative Albumin-to-Fibrinogen Ratio in Patients with Bladder Cancer. Journal of Cancer, 12(19), 5864-5873. https://doi.org/10.7150/jca.61068

30. Liu, K., Xu, Z., Meng, J., Fu, G., Gu, S., & Gu, M. (2015). Impact of preoperative nutritional risk on complications after radical cystectomy [Article]. Beijing da xue xue bao. Yi xue ban = Journal of Peking University. Health sciences, 47(5), 800-803. https://www.scopus.com/inward/record.uri?eid=2-s2.0-84955347739&partnerID=40&md5=f57b55f63f970b6e31c6701a4576c250

31. Lonati, C., & Moschini, M. (2021). Editorial Comment from Dr Lonati and Dr Moschini to Impact of preoperative sarcopenia and myosteatosis on prognosis after radical cystectomy in patients with bladder cancer [Note]. International Journal of Urology, 28(7), 762-763. https://doi.org/10.1111/iju.14577

32. Lyon, T., Turner, R., McBride, D., Gingrich, J., Davies, B., Jacobs, B., & Tarin, T. (2016). A pilot study of high-arginine nutritional supplementation prior to radical cystectomy [Journal article; Conference proceeding]. Canadian urological association journal, 10(9-10), S158-S159. https://www.cochranelibrary.com/central/doi/10.1002/central/CN-01408400/full

33. Lyon, T. D., Turner, R. M., McBride, D., Wang, L., Gingrich, J. R., Hrebinko, R. L., Jacobs, B. L., Davies, B. J., & Tarin, T. V. (2017). Preoperative immunonutrition prior to radical cystectomy: a pilot study. Canadian Journal of Urology, 24(4), 8895-8901. <Go to ISI>://WOS:000408303200005

34. Matulewicz, R., Aggarwal, A., Kim, J., & Kundu, S. (2015). PREOPERATIVE NUTRITIONAL STATUS AS A PREDICTOR OF RADICAL CYSTECTOMY POST OPERATIVE COMPLICATIONS: A NATIONAL COMPARISON. Journal of urology, 193(4), E800-E800. https://doi.org/10.1016/j.juro.2015.02.2317

35. Munbauhal, G., Drouin, S. J., Mozer, P., Colin, P., Phé, V., Cussenot, O., & Rouprêt, M. (2014). Malnourishment in bladder cancer and the role of immunonutrition at the time of cystectomy: An overview for urologists [Review]. BJU International, 114(2), 177-184. https://doi.org/10.1111/bju.12529

36. Nct. (2011). Multimodal Rehabilitation Program to Bladder Cancer Patients [Trial registry record]. https://clinicaltrials.gov/show/NCT01329107. https://www.cochranelibrary.com/central/doi/10.1002/central/CN-01532807/full

37. Nct. (2013). Prehabilitation to Enhance Postoperative Functional Capacity Following Radical Cystectomy [Trial registry record]. https://clinicaltrials.gov/show/NCT01836978. https://www.cochranelibrary.com/central/doi/10.1002/central/CN-01541729/full

38. Nct. (2017). Trimodal Prehabilitation for Cystectomy Patients to Enhance Post-operative Care [Trial registry record]. https://clinicaltrials.gov/show/NCT03347045. https://www.cochranelibrary.com/central/doi/10.1002/central/CN-01565943/full

39. Nct. (2019). Against All Odds -Prehabilitation in Urologic Cancer Surgery [Trial registry record]. https://clinicaltrials.gov/show/NCT04088968. https://www.cochranelibrary.com/central/doi/10.1002/central/CN-01984260/full

40. Nct. (2022). EffectiveNess of a Multimodal preHAbilitation Program in Patients With Muscle iNvasive Bladder canCEr [Trial registry record]. https://clinicaltrials.gov/show/NCT05480735. https://www.cochranelibrary.com/central/doi/10.1002/central/CN-02424842/full

41. Nct. (2023). The Role of Preoperative Immunonutrition on Morbidity and Immune Response After Cystectomy (INCyst Trial) [Trial registry record]. https://clinicaltrials.gov/show/NCT05726786. https://www.cochranelibrary.com/central/doi/10.1002/central/CN-02524630/full

42. Patel, S. Y., Trona, N., Alford, B., Laborde, J. M., Kim, Y., Li, R. G., Manley, B. J., Gilbert, S. M., Sexton, W. J., Spiess, P. E., & Poch, M. A. (2022). Preoperative immunonutrition and carbohydrate loading associated with improved bowel function after radical cystectomy. Nutrition in Clinical Practice, 37(1), 176-182. https://doi.org/10.1002/ncp.10661

43. Peng, L., Du, C. X., Meng, C. Y., Li, J. Z., You, C. Y., Li, X. H., Zhao, P., Cao, D. H., & Li, Y. X. (2021). Controlling Nutritional Status Score Before Receiving Treatment as a Prognostic Indicator for Patients With Urothelial Cancer: An Exploration Evaluation Methods. Frontiers in Oncology, 11, Article 702908. https://doi.org/10.3389/fonc.2021.702908

44. Poinas, G., Blache, J. L., Kassab-Chahmi, D., Evrard, P. L., Artus, P. M., Alfonsi, P., Rébillard, X., Beaussier, M., Cerantola, Y., Coloby, P., Drapier, É., Houédé, N., Masson-Lecomt, A., Rouprêt, M., Le Normand, L., Gamé, X., Bosset, P. O., Delaunay, L., Fendler, J. P., . . . Cuvelier, G. (2019). Short version of recommendations for enhanced recovery program (ERP) for cystectomy: Technical measures [Short survey]. Progres en Urologie, 29(2), 63-75. https://doi.org/10.1016/j.purol.2018.12.002

45. Pruthi, R. S., Chun, J., & Richman, M. (2003). Reducing time to oral diet and hospital discharge in patients undergoing radical cystectomy using a perioperative care plan [Article]. Urology, 62(4), 661-665. https://doi.org/10.1016/S0090-4295(03)00651-4

46. Pruthi, R. S., & Smith, A. M. (2011). Effect of Preoperative Nutritional Deficiency on Mortality After Radical Cystectomy for Bladder Cancer EDITORIAL COMMENT. Journal of urology, 185(1), 96-96. https://doi.org/10.1016/j.juro.2010.09.139

47. Smelser, W. W., Tallman, J. E., Gupta, V. K., Al Awamlh, B. A., Johnsen, N. V., Barocas, D. A., Kline-Quiroz, C., Tomlinson, C. A., McEvoy, M. D., Hamilton-Reeves, J., & Chang, S. S. (2023). Implementation of a comprehensive prehabilitation program for patients undergoing radical cystectomy. Urologic Oncology-Seminars and Original Investigations, 41(2). https://doi.org/10.1016/j.urolonc.2022.10.017

48. Smith, J., Pruthi, R. S., & McGrath, J. (2014). Enhanced recovery programmes for patients undergoing radical cystectomy [Review]. Nature Reviews Urology, 11(8), 437-444. https://doi.org/10.1038/nrurol.2014.164

49. Sung, L. H., & Yuk, H. D. (2021). Enhanced recovery after surgery of patients undergoing radical cystectomy for bladder cancer [Review]. Translational Andrology and Urology, 9(6), 2986-2996. https://doi.org/10.21037/tau.2020.03.44

50. Thoft Jensen, B. (2016). Preoperative Nutritional Status and The Impact on Radical Cystectomy Recovery: An International Comparative Study. Urologic Nursing, 36(3), 133-152. https://doi.org/10.7257/1053-816X.2016.36.3.133

JBI Critical Appraisal Tools

Tables S1-S9: JBI Checklist available at: https://jbi.global/critical-appraisal-tools

Score (mean): According to Pimsen et al., studies with a JBI score above 70% are considered of high quality, those scoring between 50% and 70% are of medium quality, and those with a score below 50% are categorized as low quality. The assessment of quality and risk of bias should be integrated as a separate column in the data extraction table to identify the appropriate level (low, medium, high).

Table S1

STUDY	ITEM 1	ITEM 2	ITEM 3	ITEM 4	ITEM 5	ITEM 6	ITEM 7	ITEM 8	ITEM 9	ITEM 10	ITEM 11	ITEM 12	ITEM 13	SCORE (mean)	SEEK FURTHER INFO	QUALITY
Jill et., al. 2018	Y	Y	Y	Y	Y	U	Y	Y	Y	Y	Y	Y	Y	92.31%		HIGH
D.Lyon et., al. 2017	N	N	Y	N	N	N	Y	Y	Y	Y	Y	Y	Y	61.54%		MEDIUM

Table S1. JBI Critical appraisal tool for Randomized Controlled Trials

Legend: Y=Yes; N=No; U=Unclear; NA=Not Applicable / Items from JBI Critical appraisal tool for Randomized Controlled Trials: 1.Was true randomization used for assignment of participants to treatment groups?; 2.Was allocation to treatment groups concealed?;3.Were treatment groups similar at the baseline?; 4. Were participants blind to treatment assignment?;5.Were those delivering treatment blind to treatment assignment? ;6.Were outcomes assessors blind to treatment assignment?;7.Were treatment groups treated identically other than the intervention of interest?;8.Was follow up complete and if not, were differences between groups in terms of their follow up adequately described and analyzed?;9.Were participants analyzed in the groups to which they were randomized?;10.Were outcomes measured in the same way for treatment groups?;11.Were outcomes measured in a reliable way?;12.Was appropriate statistical analysis used?;13.Was the trial design appropriate, and any deviations from the standard RCT design (individual randomization, parallel groups) accounted for in the conduct and analysis of the trial?

Table S2

STUDY	ITEM 1	ITEM 2	ITEM 3	ITEM 4	ITEM 5	ITEM 6	ITEM 7	ITEM 8	ITEM 9	SCORE (mean)	SEEK FURTHER INFO	QUALITY
Bente et., al. 2019	Y	U	N	N	Y	Y	Y	Y	Y	66.6%		MEDIUM

Table S2. JBI Critical appraisal tool for quasi-experimental studies

Legend: Y=Yes; N=No; U=Unclear; NA=Not Applicable / Items from JBI Critical appraisal tool for quasi-experimental studies:1=Is it clear in the study what is the 'cause' and what is the 'effect' (i.e. there is no confusion about which variable comes first)?; 2=Were the participants included in any comparisons similar?; 3=Were the participants included in any comparisons receiving similar treatment/care, other than the exposure or intervention of interest?; 4=Was there a control group?; 5=Were there multiple measurements of the outcome both pre and post the intervention/exposure?; 6=Was follow up complete and if not, were differences between groups in terms of their follow up adequately described and analyzed?; 7=Were the outcomes of participants included in any comparisons measured in the same way?;8=Were outcomes measured in a reliable way?; 9=Was appropriate statistical analysis used?

Table S3

STUDY	ITEM 1	ITEM 2	ITEM 3	ITEM 4	ITEM 5	ITEM 6	ITEM 7	ITEM 8	INCLUDE / EXCLUDE	SCORE (mean)	SEEK FURTHER INFO	QUALITY
Patel et., al. 2022	Y	Y	Y	Y	Y	Y	Y	Y	**Y**	100%		HIGH

Table S3. JBI Critical appraisal tool of analytical cross sectional studies

Legend: Y=Yes; N=No; U=Unclear; NA=Not Applicable / Items from Critical appraisal of analytical cross sectional studies: 1= Were the criteria for inclusion in the sample clearly defined?; 2=Were the study subjects and the setting described in detail?, 3=Was the exposure measured in a valid and reliable way?, 4=Were objective, standard criteria used for measurement of the condition?, 5=Were confounding factors identified?, 6=Were strategies to deal with confounding factors stated?, 7=Were the outcomes measured in a valid and reliable way?, 8=Was appropriate statistical analysis used?

Table S4

STUDY	ITEM 1	ITEM 2	ITEM 3	ITEM 4	ITEM 5	ITEM 6	ITEM 7	ITEM 8	ITEM 9	ITEM 10	SCORE (mean)	SEEK FURTHER INFO	QUALITY
Naif et. al. 2020	Y	Y	Y	Y	Y	N	N	Y	Y	Y	80%		HIGH

Table S4. JBI Critical appraisal tool for case control studies

Legend: Y=Yes; N=No; U=Unclear; NA=Not Applicable / Items from JBI Critical appraisal tool for case control studies: 1.Were the groups comparable other than the presence of disease in cases or the absence of disease in controls?; 2.Were cases and controls matched appropriately?; 3.Were the same criteria used for identification of cases and controls?; 4.Was exposure measured in a standard, valid and reliable way?; 5.Was exposure measured in the same way for cases and controls?; 6.Were confounding factors identified?; 7.Were strategies to deal with confounding factors stated?; 8.Were outcomes assessed in a standard, valid and reliable way for cases and controls?; 9.Was the exposure period of interest long enough to be meaningful?; 10.Was appropriate statistical analysis used?

Table S5

STUDY	ITEM 1	ITEM 2	ITEM 3	ITEM 4	ITEM 5	ITEM 6	ITEM 7	ITEM 8	ITEM 9	ITEM 10	ITEM 11	SCORE (mean)	SEEK FURTHER INFO	QUALITY
Cozzi, et., al. 2021	Y	Y	Y	N	N	Y	Y	Y	Y	NA	Y	72.73%		HIGH

Table S5. Critical appraisal of cohort studies included

Legend: Y=Yes; N=No; U=Unclear; NA=Not Applicable; / Items from JBI Critical appraisal checklist for Cohort Study: 1= Were the two groups similar and recruited from the same population? 2= Were the exposures measured similarly to assign people to both exposed and unexposed groups? 3= Was the exposure measured in a valid and reliable way? 4= Were confounding factors identified? 5= Were strategies to deal with confounding factors stated? 6= Were the groups/participants free of the outcome at the start of the study (or at the moment of exposure)? 7= Were the outcomes measured in a valid and reliable way? 8= Was the follow-up time reported and sufficient to be long enough for outcomes to occur? 9= Was follow-up complete, and if not, were the reasons to loss to follow up described and explored? 10= Were strategies to address incomplete follow-up utilized? 11= Was appropriate statistical analysis used?
